# Period Prevalence of COVID-19 and Influenza Symptoms in 2022 Among Adults in Jazan, Saudi Arabia

**DOI:** 10.7759/cureus.45143

**Published:** 2023-09-12

**Authors:** Ibrahim M Gosadi, Raghad Hakami, Basem Zogel, Walaa H Algadhi, Manar S Hakami

**Affiliations:** 1 Department of Family and Community Medicine, Faculty of Medicine, Jazan University, Jazan, SAU

**Keywords:** saudi arabia, jazan, respiratory symptoms, covid-19, : influenza

## Abstract

Background: Seasonal influenza and the circulating new variants of coronavirus disease 2019 (COVID-19) were predicted to increase the risk of developing respiratory symptoms in 2022. The objective of this study was to estimate the period prevalence of respiratory symptoms associated with COVID-19 and influenza among adults in 2022.

Methods: This cross-sectional study targeted adults in Jazan, Saudi Arabia. Data collection was performed in February 2023 and utilized an online approach. The assessment was performed via a self-administered questionnaire, which measured demographics, vaccination practices against COVID-19 and influenza, and the occurrence of respiratory symptoms in 2022.

Results: This study involved 625 participants, 65% of whom were women with a mean age of 23 years. Only 80 participants (13%) reported receiving the influenza vaccine in 2022. The most frequently reported symptoms were headache (55%), sore throat (43.7%), and fever (43%). Nearly 34% were diagnosed with influenza, 17% were diagnosed with COVID-19, and 8% reported coinfection of both diseases. Sixty-one percent reported the occurrence of symptoms more than once in 2022. The presence of a higher frequency of respiratory symptoms was associated with gender, age, social status, employment, asthma, and obesity (P < 0.05).

Conclusions: A majority of the participants reported multiple occurrences of respiratory symptoms in 2022. The uptake of the influenza vaccine was low in the same year, suggesting a need for a targeted approach to enhance vaccination rates among vulnerable groups.

## Introduction

Coronavirus disease 2019 (COVID-19) and influenza are common respiratory diseases that are vaccine-preventable. COVID-19 infections were first announced in China at the end of 2019, which was followed by an announcement of the disease as a public health emergency of international concern (PHEIC) in January 2020 [1,2]. After nearly three years of COVID-19 being classified as a global pandemic, the World Health Organization (WHO) announced that COVID-19 is no longer a PHEIC, but it remains an ongoing health issue that requires ongoing surveillance and application of relevant prevention and control measures [3].

Influenza is a respiratory infection that has a seasonal incidence, especially during winter. Though influenza is a self-limiting disease, it can have serious respiratory complications and can lead to fatalities among high-risk groups [4]. The severity of influenza has been reported to be higher among children under five, the elderly, people with chronic comorbidities, and pregnant women [5].

Receiving the influenza vaccine once annually is recommended due to waning immunity against the disease, which is caused by the evolving of influenza viruses [4,5]. The uptake of vaccines against COVID-19 was initiated in 2021, requiring three doses at six-month intervals to reach protective immunity against the disease [6,7]. COVID-19 vaccines have been reported to prevent serious complications of the disease and to reduce the risk of hospitalization [8]; nonetheless, their real-world effectiveness has been reported to wane over time, leading to breakthrough infections [9]. The waning protection of COVID-19 vaccines against the infection is associated with the debate on whether governments should approve the uptake of COVID-19 vaccines on an annual basis as a routine vaccination [10].

COVID-19 and influenza have similar routes of infection and similar clinical presentation [11]. Furthermore, definitive diagnoses of the diseases can only be made by laboratory testing. COVID-19 and influenza can occur simultaneously, leading to serious clinical presentation [12]. The risk of acquiring COVID-19 is still present due to the appearance of new variants of the virus, including Alpha, Beta, Gamma, Delta, and Omicron, against which the developed vaccines showed variable effectiveness [13]. Similarly, protection against influenza infection has been reported to be less optimum due to factors associated with low uptake of the vaccine on an annual basis, especially among vulnerable groups [14].

The similarity between COVID-19 and influenza and the risk of concurrent infection indicates the importance of monitoring seasonal infection rates and the application of relevant preventive measures. In a study utilizing the incidence of influenza-like illnesses in the Netherlands before and after the COVID-19 pandemic, the association between the incidence of COVID-19 and influenza-like illnesses was assessed, and it was concluded that the seasonal patterns of both infections are highly similar [15]. In addition, being infected with COVID-19 has been reported to prolong seasonal influenza symptoms and recovery time [16].

Limited evidence is available concerning the incidence of respiratory symptoms related to COVID-19 and influenza, especially after the lifting of COVID-19 precautionary measures. This study aims to estimate the period prevalence of respiratory symptoms associated with COVID-19 and influenza among a sample of adult participants in Jazan, Saudi Arabia, in 2022. In addition, the study aims to assess the association between the presence of respiratory symptoms in 2022 and the measured demographic and clinical characteristics of the sample.

## Materials and methods

Study context

This study was a cross-sectional investigation in which the identification and recruitment of participants were conducted in online settings. This study targeted adult subjects in the Jazan region in the southwest of Saudi Arabia. Data collection was performed in February 2023. Ethical approval to conduct the study was granted by the Standing Committee for Scientific Research of Jazan University (approval number: REC-44/06/474, dated 5 January 2023). Data collection was anonymous, and no identification data was collected from the participants. Data collection was initiated after an online study information sheet was provided to the participants and their informed consent was secured.

Data collection tool

An online self-administered questionnaire was utilized in the current investigation. The questionnaire was composed of three main sections. The first section measured the demographic characteristics of the participants, their smoking history, and their diagnoses with chronic diseases. The second section assessed their vaccination history against COVID-19 and influenza. The third section assessed the occurrence of respiratory symptoms related to COVID-19 and influenza, the incidence of these symptoms in 2022, and whether they were diagnosed with COVID-19 or influenza in 2022. Symptoms related to COVID-19 and respiratory symptoms were adopted from the relevant literature on the clinical presentation of the diseases [17,18]. The list of symptoms included the following: fever, cough, dyspnea, myalgia, sputum, nasal congestion, night sweat, headache, bloody cough, anosmia, confusion, rhinorrhea, chest pain, sore throat, sneezing, diarrhea, ageusia, and snoring.

Data collection process

The questionnaire was transformed into an electronic format utilizing Google Forms (Google LLC, Mountain View, California, United States). A web link was generated to facilitate the distribution of the questionnaire and access of the participants. Afterward, identification of the subjects and the approaching process were achieved via distributing the questionnaire on relevant social media platforms. A study information sheet was displayed with the generated web link describing the study objectives and the targeted population.

Adults living in the Jazan region were included. Subjects who were younger than 18 years or who were not living in Jazan were excluded. Those who agreed to participate were directed toward the questionnaire. All who were approached were asked to further distribute the questionnaire among their acquaintances; in this way, the study utilized a convenient, non-random, snowballing sampling strategy. Evidence concerning the incidence or prevalence of seasonal influenza infections in the Jazan region is currently lacking; therefore, a study assessing the seroprevalence of COVID-19 among the community in Jazan was utilized to estimate the required sample size of the current investigation. According to Alhazmi et al., the prevalence of individuals with positive COVID-19 in the Jazan region in 2020 reached 26% [19]. Therefore, assuming that 26% of the population in Jazan is at risk of developing respiratory symptoms and utilizing the StatCalc component of Epi Info™ (Centers for Disease Control and Prevention, Atlanta, Georgia, United States), with a 5% margin of error and a 99% confidence interval, a sample of 510 participants was required for the current investigation.

Data analysis

Data analysis was conducted using the IBM SPSS Statistics for Windows, Version 26.0 (Released 2019; IBM Corp., Armonk, New York, United States). Frequencies and proportions were used to summarize binary and categorical variables. Means, standard deviations, medians, and interquartile ranges were used to summarize continuous variables according to their distribution. Assessment of the occurrence of respiratory symptoms in 2022 according to the sample characteristics was tested via a chi-squared test or Fisher’s exact test.

To enable cross-tabulation, continuous variables were transformed into either binary or categorical variables according to the estimated average or relevant cut-off points. The incidence of symptoms was transformed to fit into three categories according to the estimated median of reported respiratory symptoms. The median number of reported respiratory symptoms in 2022 among the recruited sample was four symptoms, leading to categorizing the sample as having either no symptoms, one to four symptoms, or more than four symptoms. Age was transformed into a binary variable using the median age of 23 as a cut-off point. Furthermore, BMI was transformed into a binary variable using 25 as a cut-off point. Finally, to avoid the presence of empty cells when performing cross-tabulations, social status was grouped into married or not married, education into lower than a university education or university education, employment into employed or unemployed, and income into two categories with 5,000 Saudi Arabian Riyal (SAR) as a cut-off point. A p-value of less than 0.05 was designated as a statistically significant value for the applied statistical tests.

## Results

A total of 625 participants completed the recruitment process of the current investigation. The characteristics of the sample, their smoking practice, and their diagnosed conditions are displayed in Table 1. The median age of the participants was 23 (IQR: 21-38). A majority of the sample were female (65.1%), were single (60%), had an income of less than 10,000 SAR (64%), had a university education (76%), and lived in owned housing (90%). Nearly half of the sample were students, and living in an urban area. When the participants were asked about their smoking history, nearly 10% reported being current smokers, while 23.8% of the sample reported being exposed to second-hand smoking. Finally, the most frequently diagnosed conditions among the sample were obesity (12%), hypertension (7%), and diabetes (6%).

**Table 1:** Demographic characteristics, smoking history, and diagnosed chronic conditions among 625 adult participants in Jazan, Saudi Arabia. SAR: Saudi Arabia Riyal

Variable	Frequency (proportion)
Gender	
Male	218 (34.9%)
Female	407 (65.1%)
Income	
5000 SAR or less	267 (42.7%)
Between 5000 and 10000 SAR	131 (21%)
More than 10000 and less than 15000	109 (17.4%)
More than 15000	118 (18.9%)
Social Status	
Married	228 (36.5%)
Single	371 (59.4%)
Divorced	18 (2.9%)
Widowed	8 (1.3%)
Education	
Primary	3 (0.5%)
Intermediate	18 (2.9%)
Secondary	106 (17%)
University	477 (76.3%)
Postgraduate	21 (3.4%)
Employment	
Student	318 (50.9%)
Governmental	151 (24.2%)
Private	35 (5.6%)
Military	8 (1.3%)
Business Owner	2 (.3%)
Housewife	46 (7.4%)
Unemployed	37 (5.9%)
Retired	28 (4.5%)
Housing	
Owned Apartment	286 (45.8%)
Owned Villa	281 (45%)
Rented	58 (9.3%)
Location	
City	313 (50.1%)
Village	312 (49.9%)
Smoking history	
Current	58 (9.3%)
Previous	22 (3.5%)
2^nd^ hand smoking	149 (23.8%)
Never	396 (63.4%)
Diagnosed chronic conditions	
Diabetes	38 (6.1%)
Hypertension	42 (6.7%)
Asthma	35 (5.6%)
Dyslipidemia	17 (2.7%)
Obesity	73 (11.7%)
Heart disease	5 (0.8%)
Cancer	2 (0.3%)
Reported pregnancy in 2022	18 (2.9%)

Table 2 displays the vaccination practices against COVID-19 and influenza of the recruited sample. All of the sample except five participants (99%) reported receipt of a COVID-19 vaccine. In contrast, less than half of the sample (48%) reported having received the influenza vaccine in the past, and nearly 45% reported receipt of the influenza vaccine only once in their life. A majority of the sample (78%) reported receipt of three doses of a COVID-19 vaccine, with the most frequently taken vaccine being Pfizer, followed by AstraZeneca. When asked whether they had received the influenza vaccine in 2022, only 80 participants reported they had, with 51 of them receiving it between October and December.

**Table 2:** Vaccination practices against COVID-19 and influenza among 625 adult participants in Jazan, Saudi Arabia. COVID-19: coronavirus disease 2019

Variable	Frequency (proportion)
Receipt of COVID-19 vaccine	620 (99.2%)
Number COVID-19 vaccines	
One	9 (1.5%)
Two	119 (19.2%)
Three	487 (78.5%)
More than three	5 (8%)
Name of the COVID-19 vaccine in the first dose	
Pfizer	466 (75.2%)
AstraZeneca	136 (21.9%)
Moderna	12 (1.9%)
I do not remember/ Did not take the dose	6 (1%)
Name of the COVID-19 vaccine in the second dose	
Pfizer	429 (69.2%)
AstraZeneca	113 (18.2%)
Moderna	61 (9.8%)
I do not remember/ Did not take the dose	17 (2.7%)
Name of the COVID-19 vaccine in the third dose	
Pfizer	315 (50.8%)
AstraZeneca	56 (9%)
Moderna	125 (20.2%)
I do not remember/ Did not take the dose	124 (20%)
Ever received the influenza vaccine	302 (48.3%)
Frequency of receiving the influenza vaccine	
1	124 (44.6%)
2	82 (29.5%)
3	51 (18.3%)
4+	20 (7.4%)
Receipt of the influenza vaccine during the current season	80 (12.8%)
Month of receiving the influenza vaccine in 2022	
January	7 (1.1%)
February	2 (0.3%)
March	0 (0%)
April	0 (0%)
May	2 (0.3%)
June	1 (0.2%)
July	3 ( 0.5%)
August	9 (1.4%)
September	5 (0.8%)
October	16 (2.6%)
November	16 (2.6%)
December	19 (3%)

Table 3 summarizes the frequency of reported respiratory symptoms related to COVID-19 and influenza among the participants in 2022. Only 130 participants (20.1%) reported not suffering from any respiratory symptoms, while 381 (61%) reported experiencing respiratory symptoms on more than one occasion in 2022. The most frequently reported symptoms were headache (55%), sore throat (43.7%), and fever (43%). When the participants were asked whether they had been diagnosed with influenza or COVID-19, nearly 34% of the sample reported being diagnosed with influenza, while only 17% reported being diagnosed with COVID-19 in 2022. Fifty participants (8%) reported being diagnosed with both diseases in 2022. Finally, when asked whether they had been affected by complications related to their COVID-19 or influenza infection following having respiratory symptoms in 2022, 23% of the participants reported being affected by pain in joints or muscles, while 17% reported having a sleep disturbance (Table 4).

**Table 3:** Frequency of experiencing respiratory symptoms in 2022 among 625 adult participants in Jazan, Saudi Arabia. COVID-19: coronavirus disease 2019

Symptom	Frequency (proportions)
Fever	269 (43%)
Myalgia	238 (38.1%)
Headache	342 (54.7%)
Sweating	77 (12.3%)
Shortness of breath	151 (24.2%)
Fatigue and weakness	205 (32.8%)
Nasal obstruction or congestion	256 (41%)
Runny nose	256 (41%)
Sore throat	273 (43.7%)
Eye pain	128 (20.5%)
Vomiting and diarrhea	74 (11.8%)
Dry cough	161 (25.8%)
Cough with sputum	152 (24.3%)
Phlegmy cough	13 (2.1%)
Sneezing	195 (31.2%)
Confusion	77 (12.3%)
Chest pain	111 (17.8%)
Snoring	70 (11.2%)
Number of incidence of these symptoms during 2022	
Once	114 (18.2%)
Twice	175 (28%)
Three times	95 (15.2%)
Four times	33 (5.3%)
More than 4 times	78 (12.5%)
None	130 (20.8%)
Confirmed diagnosis of influenza	212 (33.9%)
Confirmed diagnosis of COVID-19	103 (16.5%)
Confirmed diagnosis of influenza and COVID-19	50 (8%)

**Table 4:** Frequency of reported complications related to COVID-19 or influenza among 625 adult participants in Jazan, Saudi Arabia. COVID-19: coronavirus disease 2019

Complications	Frequency (proportion)
Inability to exercise	62 (9.9%)
Heart disease	6 (1%)
Loss of sense of smell and taste	48 (7.7%)
Clotting disease	5 (0.8%)
Cognitive impairment	70 (11.2%)
Depression or anxiety	41 (6.6%)
Sleep disturbance	109 (17.4%)
Pain in joints or muscles	142 (22.7%)
Renal failure	3 (0.5%)
Persistence of symptoms for more than 4 weeks	36 (5.8%)

The association between reported respiratory symptoms among adults in Jazan, Saudi Arabia, in 2022 and the sample characteristics are displayed in Table 5. Gender, age, social status, employment, asthma, and obesity were associated with variation in the presence of respiratory symptoms with a statistically significant difference (P < 0.05). Women were more likely to report symptoms in comparison to men. Similarly, younger participants were more likely to report symptoms in comparison to older ones. It is noted that the higher frequency of non-married and unemployed participants reporting respiratory symptoms is likely influenced by age variation within the recruited sample. In terms of the measured chronic morbidities, participants with asthma and obesity were more likely to report having respiratory symptoms in comparison to those without asthma or obesity. Finally, neither receipt of a COVID-19 vaccine nor the influenza vaccine was associated with reported respiratory symptoms among the recruited sample.

**Table 5:** Association between frequency of reported respiratory symptoms and sample characteristics among 625 adult participants in Jazan, Saudi Arabia. *Chi-squared test **Fisher’s exact test SAR: Saudi Arabia Riyal; COVID-19: coronavirus disease 2019

	Number of reported symptoms			Total	p-value
	None	One to four symptoms	More than four symptoms		
Gender					0.001*
Male	58 (26.6%)	73 (33.5%)	87 (39.9%)	218 (100%)	
Female	58 (14.3%)	149 (36.6%)	200 (49.1%)	407 (100%)	
Age					<0.001*
Less than 23	66 (22.5%)	79 (27%)	148 (50.5%)	293 (100%)	
23 or older	50 (15.1%)	143 (43.1%)	139 (41.9%)	332 (100%)	
BMI					0.132*
Less than 25	71 (21%)	111 (32.8%)	156 (46.2%)	338 (100%)	
25 or more	44 (15.4%)	110 (38.6%)	131 (46%)	285 (100%)	
Social status					0.003*
Married	34 (14.9%)	100 (43.9%)	94 (41.2%)	228 (100%)	
Non- married	82 (20.7%)	122 (30.7%)	193 (48.6%)	397 (100%)	
Education					0.228*
Less than university education	28 (22%)	49 (38.6%)	50 (39.4%)	127 (100%)	
University education	88 (17.7%]	173 (34.7%)	237 (47.6%)	498 (100%)	
Employment					0.024*
Employed	31 (16%)	84 (43.3%)	79 (40.7%)	194 (100%)	
Non-employed	85 (19.7%)	138 (32%)	208 (48.3%)	431 (100%)	
Housing					0.887*
Owned	105 (18.5%)	200 (35.3%)	262 (46.2%)	567 (100%)	
Rented	11 (19%)	22 (37.9%)	25 (43.1%)	58 (100%)	
Location					0.535*
City	59 (18.8%)	117 (37.4%)	137 (43.8%)	313 (100%)	
Village	57 (18.3%)	105 (33.7%)	150 (48.1%)	312 (100%)	
Income					0.331*
5000 SAR or less	55 (20.6%)	87 (32.6%)	125 (46.8%)	267 (100%)	
More than 5000 SAR	61 (17%]	135 (37.7%)	162 (45.3%)	358 (100%)	
Smoking					0.245**
Current	9 (15.5%)	24 (41.4%)	25 (43.1%)	58 (100%)	
Previous	7 (31.8%)	8 (36.4%)	7 (31.8%)	22 (100%)	
2^nd^ hand	20 (13.4%)	52 (34.9%)	77 (51.7%)	149 (100%)	
Never	80 (20.2%)	138 (34.8%)	178 (44.9%)	396(100%)	
Diabetes					0.381*
Yes	4 (10.5%)	14 (36.8%)	20 (52.6%)	38 (100%)	
No	112 (19.1%)	208 (35.4%)	267 (45.5%)	587 (100%)	
Hypertension					0.562*
Yes	6 (14.3%)	18 (42.9%)	18 (42.9%)	42 (100%)	
No	110 (18.9%)	204 (35%)	269 (46.1%)	583 (100%)	
Asthma					0.020*
Yes	3 (8.6%)	8 (22.9%)	24 (68.6%)	35 (100%)	
No	113 (19.2%)	214 (36.3%)	263 (44.6%)	590 (100%)	
Dyslipidemia					0.322**
Yes	2 (11.8%)	9 (52.9%)	6 (35.3%)	17 (100%)	
No	114 (18.8%)	213 (35%)	281 (46.2%)	608 (100%)	
Obesity					0.027*
Yes	6 (8.2%)	25 (34.2%)	42 (57.5%)	73 (100%)	
No	110 (19.9%)	197 (35.7%)	245 (44.4%)	552 (100%)	
Heart disease					0.851**
Yes	1 (20%)	1 (20%)	3 (60%)	5 (100%)	
No	115 (18.5%)	221 (35.6%)	284 (45.8%)	620 (100%)	
Pregnancy in 2022					0.666**
Yes	2 (11.1%)	6 (33.3%)	10 (55.6%)	18 (100%)	
No	114 (18.8%)	216 (35.6%)	277 (45.6%)	607 (100%)	
Number of received COVID-19 vaccines					0.397**
One	4 (44.4%)	2 (22.2%)	3 (33.3%)	9 (100%)	
Two	20 (16.8%)	46 (38.7%)	53 (44.5%)	119 (100%)	
Three	90 (18.5%)	170 (34.9%)	227 (46.6%)	487 (100%)	
More than three	2 (40%)	1 (20%)	2 (40%)	5 (100%)	
Ever received the flu vaccine					0.932*
Yes	58 (19.2%)	106 (35.1%)	138 (45.7%)	302 (100%)	
No	58 (18%)	116 (35.9%)	149 (46.1%)	323 (100%)	
Receipt of the flu vaccine in 2022					0.249*
Yes	16 (20%)	35 (42.5%)	30 (37.5%)	80 [100%]	
No	100 (18.3%)	188 (34.5%)	257 (47.2%)	545 [100%]	

## Discussion

This study was a cross-sectional investigation that assessed the period prevalence of respiratory symptoms related to COVID-19 and influenza among adult participants in Jazan, Saudi Arabia, in 2022. A majority of the participants reported suffering from respiratory symptoms in 2022, and only one fifth reported not experiencing any respiratory symptoms. A majority of the participants also reported experiencing respiratory symptoms on more than one occasion in 2022. The most frequently reported symptom was headache, followed by sore throat and fever. Nearly one third of the sample reported being diagnosed with influenza, while only 17% of the participants reported being diagnosed with COVID-19 in 2022. Fifty participants (8%) reported being diagnosed with both diseases in 2022. The presence of higher frequency of respiratory symptoms was associated with gender, age, social status, employment, asthma, and obesity.

The findings of the current investigation can be compared to those of similar local and international studies assessing the presence of respiratory symptoms related to COVID-19 and influenza. Nonetheless, it must be noted that studies on the prevalence of respiratory symptoms related to seasonal influenza in Saudi Arabia are currently lacking. A majority of studies assessing seasonal influenza in Saudi Arabia are related to vaccination uptake rather than infection rates or the presence of respiratory symptoms [14,20-26]. Nonetheless, studies assessing symptoms related to COVID-19 among the Saudi population revealed comparable findings.

In a study by Gosadi et al., which recruited a sample of 1,026 individuals in Jazan, Saudi Arabia, to assess clinical presentation related to COVID-19 infection, it was reported that the most frequently reported clinical presentations were related to fever and headache [27]. They also reported that patients diagnosed with asthma were more likely to suffer from respiratory symptoms related to COVID-19. Both these above are similar to the findings of the current study. However, Gosadi et al. identified a statistically significant variation in the distribution of COVID-19 symptoms according to smoking, where no such association was detected in the current study.

Similar international investigations on the prevalence of influenza-like symptoms and COVID-19 are limited. In a study by Jha et al., which recruited a sample of 3,667 healthcare workers from India to assess the prevalence of influenza-like symptoms and COVID-19 in 2020, it was revealed that 14.7% had influenza-like symptoms and that only 20 participants had tested positive for an influenza virus [28]. Our study identified a higher prevalence of influenza-related symptoms and a higher proportion of individuals diagnosed with influenza. This variation can be partially explained by the variation in the targeted population and the difference in the assessment periods. Nonetheless, Jha et al. did not report the most frequently reported respiratory symptoms among their sample.

Our study identified an association between obesity and higher frequency of respiratory illness. This is supported by a systematic review and meta-analysis that was performed to assess the association between obesity and severity of influenza and COVID-19, which indicated that obesity can be associated with a higher risk of severe morbidity and mortality for both influenza and COVID-19 [29].

The current study identified a high prevalence of individuals who reported the occurrence of respiratory symptoms more than once in 2022. No similar previous studies that assessed the recurrent incidence of respiratory symptoms before 2022 could be identified to enable an evaluation of whether 2022 witnessed a high relapse of respiratory infections in comparison to previous years. Nonetheless, a higher incidence of respiratory illness due to coinfection with COVID-19 and influenza was expected [30], suggesting that COVID-19 surveillance should be incorporated into existing influenza surveillance systems [31].

The Saudi Ministry of Health declared that seasonal influenza in 2022 was likely to be more severe in comparison to the previous two years [32] and urged the public, especially vulnerable groups such as those with asthma, to receive the vaccine. This is supported by the findings of the WHO’s influenza weekly update reports, between week 27 of 2021 and week 13 of 2023. The number of positive specimens for influenza in the Eastern Mediterranean region was higher in 2022 in comparison to 2021 [33].

The current study identified high vaccination rates with the COVID-19 vaccine but not the influenza vaccine. The higher rates of COVID-19 vaccine can be explained by the fact that COVID-19 vaccination was highly encouraged in Saudi Arabia and was even mandated on some occasions [34], with more than 68 million vaccine doses administered as of April 2023 [35]. Nonetheless, less than half of the participants in the current study reported having received the influenza vaccine in the past, and only 80 participants (13%) reported receiving the vaccine in 2022. The low uptake rate of the influenza vaccine revealed in the current investigation is similar to other reports in Saudi Arabia [14,20-26].

This study has both strengths and limitations. This study utilized an online approach for data collection, which enabled an assessment of the distribution of respiratory symptoms among a sample with variable demographic characteristics. In addition, using this approach facilitated reaching a sample with respiratory symptoms who have not attended healthcare facilities for management of their symptoms. Nonetheless, using an online method for data collection increases the probability of the study not recruiting subjects who cannot read and write. Furthermore, this study relied on the participants recalling the occurrence of their symptoms due to the retrospective nature of the investigation, which may make the measurement subject to recall bias. Nonetheless, the recall period is relatively short, which may reduce the effect of this bias. Finally, although some participants reported being diagnosed either with COVID-19, influenza, or both during the assessment period, it was not possible to confirm whether the occurrence of respiratory symptoms among those who were not diagnosed was caused by COVID-19, influenza, or by other similar respiratory pathogens. 

## Conclusions

A majority of the participants reported suffering from respiratory symptoms and reported the occurrence of symptoms more than once in 2022. The most frequently reported symptom was headache, followed by sore throat and fever. Nearly one-third of the sample reported being diagnosed with influenza, and nearly one-fifth reported being diagnosed with COVID-19, while some indicated concurrent diagnoses with both diseases. While vaccination rates against COVID-19 were high, uptake of the influenza vaccine was very low in 2022. Higher reporting of symptoms was identified among participants with asthma and obesity, which indicates that promoting the influenza vaccine among vulnerable groups should incorporate targeted approaches. The findings of the study suggest the need to perform a prospective follow-up investigation involving clinical assessments to provide a better evaluation of the incidence of respiratory illness in the Jazan region.
